# Engineering bioactive surfaces on nanoparticles and their biological interactions

**DOI:** 10.1038/s41598-020-75465-z

**Published:** 2020-11-12

**Authors:** Meghana Matur, Harishkumar Madhyastha, T. S. Shruthi, Radha Madhyastha, S. P. Srinivas, P. N. Navya, Hemant Kumar Daima

**Affiliations:** 1 Nano-Bio Interfacial Research Laboratory (NBIRL), Department of Biotechnology, Siddaganga Institute of Technology, Tumkur, Karnataka 572103 India; 2 Department of Applied Physiology, Faculty of Medicine, University of Miyazaki, Miyazaki, Miyazaki 8891692 Japan; 3 School of Optometry, Indiana University, Bloomington, IN 47405 USA; 4 Department of Biotechnology, Bannari Amman Institute of Technology, Sathyamangalam, Erode, Tamil Nadu 638401 India; 5 Amity Center for Nanobiotechnology & Nanomedicine (ACNN), Amity Institute of Biotechnology, Amity University Rajasthan, Kant Kalwar, NH-11C, Jaipur-Delhi Highway, Jaipur, Rajasthan 303002 India

**Keywords:** Biotechnology, Chemical biology

## Abstract

The successful integration of nanoparticles into biomedical applications requires modulation of their surface properties so that the interaction with biological systems is regulated to minimize toxicity for biological function. In the present work, we have engineered bioactive surfaces on gold (Au) and silver (Ag) nanoparticles and subsequently evaluated their interaction with mouse skin fibroblasts and macrophages. The Au and Ag nanoparticles were synthesized using tyrosine, tryptophan, isonicotinylhydrazide, epigallocatechin gallate, and curcumin as reducing and stabilizing agents. The nanoparticles thus prepared showed surface corona and exhibited free radical scavenging and enzyme activities with limited cytotoxicity and genotoxicity. We have thus developed avenues for engineering the surface of nanoparticles for biological applications.

## Introduction

In the field of nanotechnology, metal nanoparticles have gathered attention because of their novel physicochemical properties and potential biological applications. Thus, metal nanoparticles have found their applicability in targeted drug and gene delivery, biosensing, dietary supplements, microbial management, wound healing, surface-enhanced Raman scattering (SERS) imaging, plasmonic photothermal therapy (PPT), and medical implants^1–8^. Numerous methods have been proposed to synthesize metal nanoparticles with desirable physicochemical properties and enhanced biomedical profiles. However, they have employed chemical reduction methods, which are not only unstable but entail safety concerns^9,10^. Nevertheless, the undesirable effects of chemical reduction methods can be circumvented by using biomolecules as reducing and stabilizing agents. In this regard, amino acids, biocompatible polymers^11–13^, monosaccharides, and sucrose have been employed in the preparation of metal nanoparticles^14^. Furthermore, extracts of several plant tissues and fruits have also been employed in the preparation of metal nanoparticles. Such extracts contain alkaloids, co-enzymes, phenolic compounds, terpenoids, and other plant metabolites that can serve as reducing agents^15–20^. Furthermore, plants, algae, fungi, bacteria, and viruses serve as low-cost raw materials/sources as nanoparticles^21–25^.

**Figure 1:**
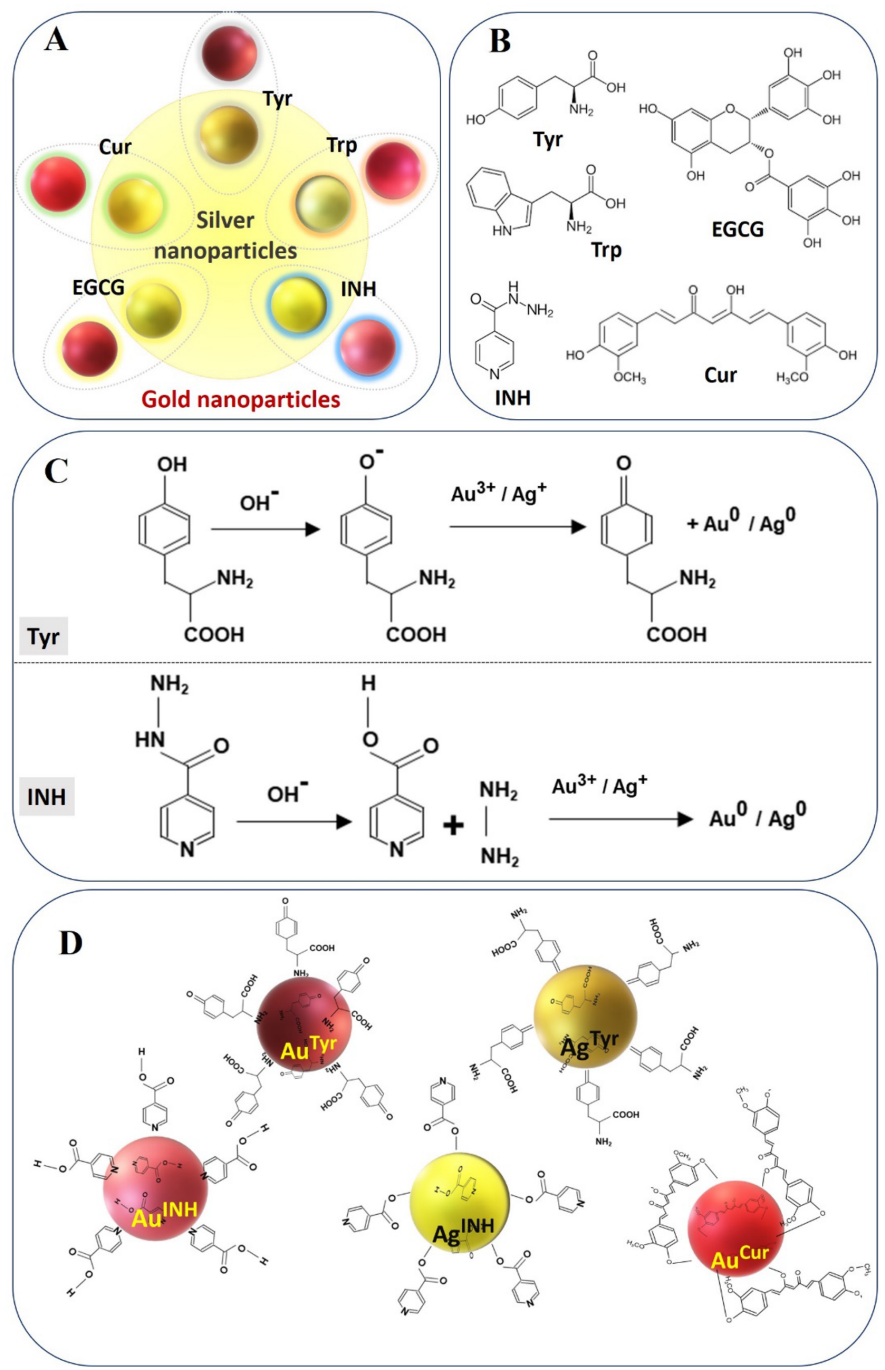
Au and Ag nanoparticles and their surface corona: (**A**) Schematic representation of Tyr, Trp, INH, EGCG, and Cur on Au nanoparticles (outside the circle) and Ag nanoparticles (inside the circle). (**B**) Biomolecules (reducing agents) employed to confer surface corona on the nanoparticles. (**C**) Mechanism of reduction of metal ions by Tyr and INH. (**D**) The molecular orientation of Tyr, INH, and Cur on the surface of Au or Ag nanoparticle.

Among the metal nanoparticles, gold (Au) and silver (Ag) nanoparticles are of particular interest as they offer unique properties: (1) they are easy to prepare, (2) Au nanoparticles are biocompatible, (3) Ag nanoparticles possess antimicrobial properties, (4) synthesis of Au and Ag nanoparticles with desirable physicochemical properties is cost-effective, and (v) stability of Au and Ag nanoparticles can be enhanced by surface modifications^18,26–30^. When administered to living systems, nanoparticles bind to a variety of proteins and biomolecules, leading to the formation of a dynamic “protein or biomolecule corona”^31–34^. Such stable surface coronas determine the biological identity of the associated nanoparticles. In particular, the surface coronas have the potential to mediate interactions with the biological entities and thus govern binding, cellular internalization, biodistribution, and toxicity of the nanoparticles^35–38^. In the present study, our goal is to induce novel surface corona on Au and Ag nanoparticles using several biomolecules, including tyrosine (Tyr), tryptophan (Trp), isonicotinylhydrazide (INH), epigallocatechin gallate (EGCG), and curcumin (Cur). These molecules are of biological origin, non-toxic and capable of reducing metal ions to produce stable nanoparticles. For example, Tyr and Trp amino acids reduce gold and silver ions through their phenol and indole groups, respectively^11,30,39^; whereas, INH undergoes slow hydrolysis at alkaline conditions to produce hydrazine (H_2_N–NH_2_), which works as a reducing agent to make metal nanoparticles^26,40^. Likewise, the polyphenols present in EGCG are responsible for nanoparticle formation^41^. The pH-dependent solubility and stability of Cur drive the formation of metallic nanoparticles through its phenolate and enolate anions^42^. Moreover, following metal ions reduction, the molecules cover the Au and Ag nanoparticles (Fig. 1) and potentially impart functionality to nanoparticles. Based on this hypothesis, we have shown that certain biomolecules form a surface corona on nanoparticles, and moreover, the particles show free radical scavenging capacity (RSC), peroxidase-like enzyme activity, limited cytotoxicity, and DNA damage.

## Experimental section

### Materials

Tetrachloroauric acid > 99.995% (HAuCl_4_), silver nitrate (AgNO_3_), potassium hydroxide > 85.0% (KOH), tyrosine > 98.0%, tryptophan > 98.0%, isonicotinylhydarzide, epigallocatechin gallate > 97.0%, curcumin > 94.0%, 2,2′-azino-bis (3-ethylbenzothiazoline-6-sulphonic acid) > 98.0%, 3,3′,5,5′tetramethylbenzidine, hydrogen peroxide 30% (w/w) in H_2_O (H_2_O_2_), potassium persulfate (K_2_S_2_O_8_), trypsin, ethidium bromide (EtBr), dimethyl sulfoxide (DMSO), Triton X-100, sodium sarcosinate, ethylenediaminetetraacetic acid (EDTA), sodium chloride (NaCl), methanol, Trizma salt (Trizma salt and Trizma base), Na-EDTA, and sodium hydroxide (NaOH) were procured from Sigma Aldrich and used as received. The 99% purity molecular grade reagents and chemicals were used, and any deviation from this purity level has been stated next to the chemical. All the solutions for the synthesis of nanoparticles were prepared using ‘ultrapure’ Milli-Q water with a resistivity of 18.2 MΩ cm at 25 °C. The cell cytotoxicity studies were carried out on m5S mouse skin fibroblasts, and cells were purchased from RIKEN Cell Bank (Tsukuba, Japan). The fibroblasts were grown in a humidified chamber (5% CO_2_, 37 °C) in alpha-minimum essential medium (α-MEM) containing 10% heat-inactivated fetal bovine serum (FBS), 2 mM glutamine, 100 U/ml penicillin G, and 100 µg/ml streptomycin. The viability/cytotoxicity multiplex assay kit (CCK 8, Dojindo Molecular Technologies Inc. Kumamoto, Japan) and DNA Extraction kit (DNeasy, #6904, Qiagen Inc, Germantown, MD, USA) were acquired and employed according to manufacturer instructions. RAW 264.2 macrophage (RAW cells) were purchased from Riken cell bank ( Riken Bioresource Research Center, Tsukuba, Japan) and cultured with RPMI-1640 (Nacalai Tesque Inc. Kyoto, Japan) supplemented with 10% heat-inactivated fetal bovine serum (Funakoshi, Tokyo, Japan) and antibiotic mixture (5 mg/ml penicillin, 5 mg/ml streptomycin and 10 mg/ml neomycin (Gibco, Tokyo, Japan) under standard culture condition of 37 °C, 5% CO_2_ and 95% humidity.

### Synthesis of gold (Au) and silver (Ag) nanoparticles

The Au and Ag nanoparticles were synthesized using different reducing agents, as shown in Fig. 1A,B. Typically, 100 ml of aqueous solutions with 1 mM KOH containing 0.1 mM Tyr, Trp, INH, EGCG, or Cur, respectively, were allowed to heat at constant stirring conditions, followed by the addition of 0.1 mM [AuCl_4_]^−^ or Ag^+^ ions to obtain Au and Ag nanoparticles. Further, all these nanoparticle solutions were heated to reduce their volume to ~ 50 ml, so that the concentrations of nanoparticles are increased. All the nanoparticle solutions were found to be stable even after concentrating, which represents that the nanoparticles are capped by the corresponding reducing agents. This has further been confirmed using several techniques and discussed in later sections. Throughout the discussion, the nanoparticles of Au and Ag are symbolized by Au^Tyr^, Au^Trp^, Au^INH^, Au^EGCG^, Au^Cur^, Ag^Tyr^, Ag^Trp^, Ag^INH^, Ag^EGCG^, and Ag^Cur^, respectively based on the respective reducing agent.

### Physicochemical characterization of nanoparticles

All the nanoparticles are characterized using several techniques to understand their physicochemical nature prior to biological activities. The UV–Visible spectrophotometer (Shimadzu UV-1800) operated at a resolution of 2 nm and used to understand the SPR properties of nanoparticles. Fourier-transform infrared spectroscopy (FTIR) analysis of all the nanoparticles was performed using a Bruker spectrophotometer with a resolution of 4 cm^−1^. Transmission electron microscopy (Hitachi, HT-7700, Tokyo, Japan) was used to obtain micrographs of nanoparticles. For TEM, 1 µl of nanoparticles sample was loaded on to the copper grid and allowed to vacuum dry at room temperature. High resolution (100 K times) TEM observations were made by the Hitachi TEM system. Zeta (ζ) potential and dynamic light scattering (DLS) measurements on different nanoparticle solutions were performed using Malvern Zetasizer Ver. 7.10 (Serial number MAL1045544). The ζ potential measures the surface charge on the nanoparticles, which is essential to identify the stability of nanoparticles and their surface corona. The DLS confirms hydrodynamic radii of the nanoparticles in the solution. Atomic absorption spectroscopy (AAS) was performed on aqua regia digested nanoparticle solutions to analyze the metal concentration and was carried out at Industrial and Scientific Research Centre, Bengaluru, India.

### Assessment of radical scavenging capacity (RSC) of nanoparticles

In order to evaluate the in vitro RSC of nanoparticles, a modified ABTS assay was performed, wherein blue/green ABTS radical cations (ABTS^·+^) chromophore were directly generated through the reaction between ABTS and potassium persulfate. The introduction of an antioxidant into the pre-formed ABTS^·+^ reduces ABTS, depending upon the antioxidant activity, leading to decolorization as percentage inhibition of the ABTS^·+^^43^. 7.4 mM ABTS and 2.45 mM potassium persulfate were added to produce ABTS^·+^ and kept in the dark for 16 h. The 16 h incubation was given to a mixture of ABTS and potassium persulfate because they react stoichiometrically (at a ratio of 1:0.5), resulting in incomplete oxidation of the ABTS^43^. Nevertheless, the ABTS oxidation begins instantly. However, the absorbance maxima were stable after more than 8 h, and the stable radicals could be stored in the dark at room temperature for use within two days. The stock ABTS^·+^ solution was diluted with ethanol until an absorbance of 0.67 ± 0.02 at 734 nm was achieved^18,30,44^. The different concentrations (0.1, 0.2, 0.6, and 1.2 ppm) of nanoparticles were allowed to react with ABTS^·+^. The decrease in the absorbance was measured at 734 nm using UV–Vis spectrophotometer. The scavenging percentage of ABTS^·+^ with nanoparticles were calculated using the formula^18,30^:

$$\% RSC = \left[ {\left( {A_{c} - A_{s} } \right)/A_{c} } \right] \times 100.$$

wherein, RSC is radical scavenging capacity; A is the absorbance at 734 nm (A_c_ is the absorbance of the control and A_s_ is the absorbance of the sample). In similar experimental conditions, Tyr, Trp, INH, EGCG, Cur, [AuCl_4_]^−^ and Ag^+^ were also used for their potential RSC activities.

### Nanozyme-mimic activity

Peroxidase nanozyme-mimic action of Au and Ag nanoparticles synthesized using Tyr, Trp, INH, EGCG, and Cur was observed using a chromogenic substrate TMB by catalyzing its oxidation in the presence of H_2_O_2_ at room temperature^30,45^. In dark experimental conditions, various nanoparticles were incubated with an aqueous solution of TMB and H_2_O_2_ to evaluate the effect of surface corona and metal composition on their peroxidase-mimic activity. The conversion of the TMB substrate was measured at 650 nm in time course mode, and all the reactions were executed at room temperature. In similar experimental conditions, the reducing agents (Tyr, Trp, INH, EGCG, and Cur) and precursor metal ions ([AuCl_4_]^−^ and Ag^+^) were also employed to assess their potential peroxidase nanozyme-mimic activities.

### Cell viability and cytotoxicity assessment of nanoparticles

Multiplex cell viability/cytotoxicity assay kit was used to determine the viable cell population and dead cells. Briefly, 1 × 10^4^ cells/ml, m5S mouse skin fibroblasts were cultured in α-MEM (Nacalai Tesque Inc. Kyoto, Japan) conditioned with 10% FBS (BioSource, Rockville, MD, USA) and 1% antibacterial and antimycotic cocktail solution (Sigma-Aldrich, St. Louis, USA). Cells were cultured in the humidified chamber (5% CO_2_, 37 °C) up to the confluent stage. The cells were treated with different concentrations (0.5, 1.0, and 5.0 µg/ml) of nanoparticles for 16 h. After exposure, the cells were washed with phosphate-buffered saline (PBS), and 10 μl of the CCK-8 solution was added to each well of the plate. The plates were incubated at 37 °C in the CO_2_ incubator for 4 h, and the absorbance was measured at 490 nm (Multiskan FC, Thermo-Fisher Scientific, Inc., Pittsburg, PA, USA). The untreated sets were also run under similar experimental conditions, and the data represent the percentage (%) of viable cells in three different groups.

For cytotoxicity assessment, the cell membrane integrity was evaluated by measuring LDH release using the multiplex assay kit. The m5S mouse skin fibroblasts were seeded into 96 well plates at a density of 1 × 10^4^ cells/well, and nanoparticles (0.5, 1.0, and 5.0 µg/ml) were added to the wells, followed incubation at 37 °C for 16 h and centrifuged at 25 G for 5 min. Cell-free culture media was collected, and 100 μl of the reaction mixture was added. The cells were incubated at room temperature for 30 min in the dark to measure the amount of LDH released into the medium using a UV–visible spectrophotometer at an absorbance of 490 nm. The control experiments were conducted under similar experimental conditions, and the data represent the percentage (%) of cytotoxicity in three different test groups.

The cytotoxicity endpoint was determined by MTT assay for RAW-264.7 cells, which were cultured in 96 well culture plates (True line, Biotechnology, Germany) at a density of 1 × 10^4^ cells/ml up to sub confluence stage. The cells were then treated with various doses (0.5, 1.0, and 5.0 μg/ml) of nanoparticles, and incubated for 12 h. The control experiments were also conducted under similar experimental conditions. The intracellular purple formazan was quantified with a UV–Vis spectrophotometer at an absorbance of 570 nm (Thermo Scientific, Multiskan FC, Pittsburg, USA).

### Genotoxicity/DNA damage assessment of nanoparticles

The m5S cells were seeded in 24 well culture plates and treated with different nanoparticles of various concentrations (0.5, 1.0, and 5.0 µg/ml) and incubated for 24 h. The single-cell DNA damage assay was carried out as per the reported protocol^46^ to estimate the potential genotoxic effect of nanoparticles. After incubation, the cells were harvested with a 0.5% trypsin EDTA solution, and the total cell population of 1 × 10^6^ cells/ml was processed for DNA damage assay. The cells were suspended in 0.25% low melting agarose, and the suspension was embedded in 1% normal melting agarose coated slide in an ice-cold tray. Then, the slides were immersed in lysis solution containing 100 mM EDTA, 2.5 M NaCl, 100 mM Trizma base, 1% sodium sarcosinate (pH 10.0), 1% Triton X-100, and 10% DMSO at 4 °C for 1 h. Later the slides were subjected to alkaline treatment (1 mM Na-EDTA, 300 mM NaOH, pH 13.0), and electrophoresis was carried out for 30 min at 4 °C. After electrophoresis, the slides were neutralized with neutralizing buffer (0.5 M Trizma base-HCl, pH 7.5) and were fixed with 100% methanol. The slides were dried and then stained with EtBr. 100 cells were counted for fragmented DNA tail using a Leica fluorescent microscope (Leica, SP 8000, Germany), and results were analyzed using Comet Analyzing Software (Version 7.1.0 rev 13. April 2016, Andor, Ltd, Shinagawa, Japan, https://andor.oxinst.com/products/komet-software/komet-7) and expressed as DNA % tail (DNA tail intensity).

### DNA ladder assay

m5S cells (3 × 10^4^ cells/well) were cultured on six well plates according to standard procedure. The cells were treated with single dose test samples Au^Tyr^ (1 µg/ml), Au^Trp^ (1 µg/ml), Au^INH^ (1 µg/ml), Au^EGCG^ (5 µg/ml), Au^Cur^ (5 µg/ml), Ag^Tyr^ (5 µg/ml), Ag^Trp^ (0.5 µg/ml), Ag^INH^ (5 µg/ml), Ag^EGCG^ (5 µg/ml), Ag^Cur^ (5 µg/ml), Tyr (10.0 µg/ml), Trp (10.0 µg/ml), INH (0.1 µg/ml), EGCG (10.0 µg/ml), Cur (10.0 µg/ml), AuCl_4_^−^ (0.1 µg/ml), Ag^+^ (0.1 µg/ml) for 24 h. Cells were washed twice with cold PBS and fragmented DNA was isolated using a DNA extraction kit according to manufacturer instructions. DNA extracts were electrophoresed on 1.5% agarose gel containing EtBr at 50 V/cm for 60 min. Ladder formation of oligonucleosomal DNA was detected under ultraviolet light and photographed using LAS 4000 Image QuantTM (Fujifilm industries Ltd, Tokyo, Japan). 1 Kbp DNA ladder was used as standard reference (Nacalai Tesque Inc. Kyoto, Japan).

## Result and discussion

As shown in Fig. 1A, different types of Au and Ag nanoparticles were synthesized using Tyr, Trp, INH, EGCG, and Cur. The biomolecules behaved as reducing as well as stabilizing agents in the preparation of nanoparticles. Based on the reducing biomolecule, the nanoparticles are denoted as Au^Tyr^, Au^Trp^, Au^INH^, Au^EGCG^, Au^Cur^, Ag^Tyr^, Ag^Trp^, Ag^INH^, Ag^EGCG^, and Ag^Cur^, respectively. The chemical structure and presene of specific functional group in Tyr, Trp, INH, EGCG, and Cur is provided in Fig. 1B. The reducing functional group reduces metal ions, and post-synthesis confer biomolecular surfaces around the nanoparticles^11,26,28^. In alkaline conditions (pH 8.4–8.6), the phenol (Tyr) and indole (Trp) are responsible for reducing the metal ions of Au and Ag, leading to the formation of Au^Tyr^, Au^Trp^, Ag^Tyr^, and Ag^Trp^, respectively^11,28,47^. Recently, we have shown that the slow hydrolysis of INH at alkaline pH produces hydrazine (H_2_N–NH_2_), which effectively reduces [AuCl_4_]^−^ and Ag^+^ ions leading to the formation of Au^INH^ and Ag^INH^ nanoparticles, respectively^26,40^. On the other hand, EGCG contains bioactive polyphenol, which enabled the formation of Au^EGCG^ and Ag^EGCG^^41^. In the case of Cur, at higher pH value (pH 9.4), the production of phenolate and enolate anions starts the reduction of [AuCl_4_]^−^ and Ag^+^ ions to generate Au^Cur^ and Ag^Cur^, respectively^42^. The representative reduction of [AuCl_4_]^−^ or Ag^+^ ions from Tyr and INH moieties under alkaline reduction conditions is depicted in Fig. 1C. The molecular orientation of oxidised-Tyr, oxidised-INH, and oxidised-Cur on the surface of respective Au and Ag nanoparticles is illustrated in Fig. 1D^26,28,42,48^.

**Figure 2:**
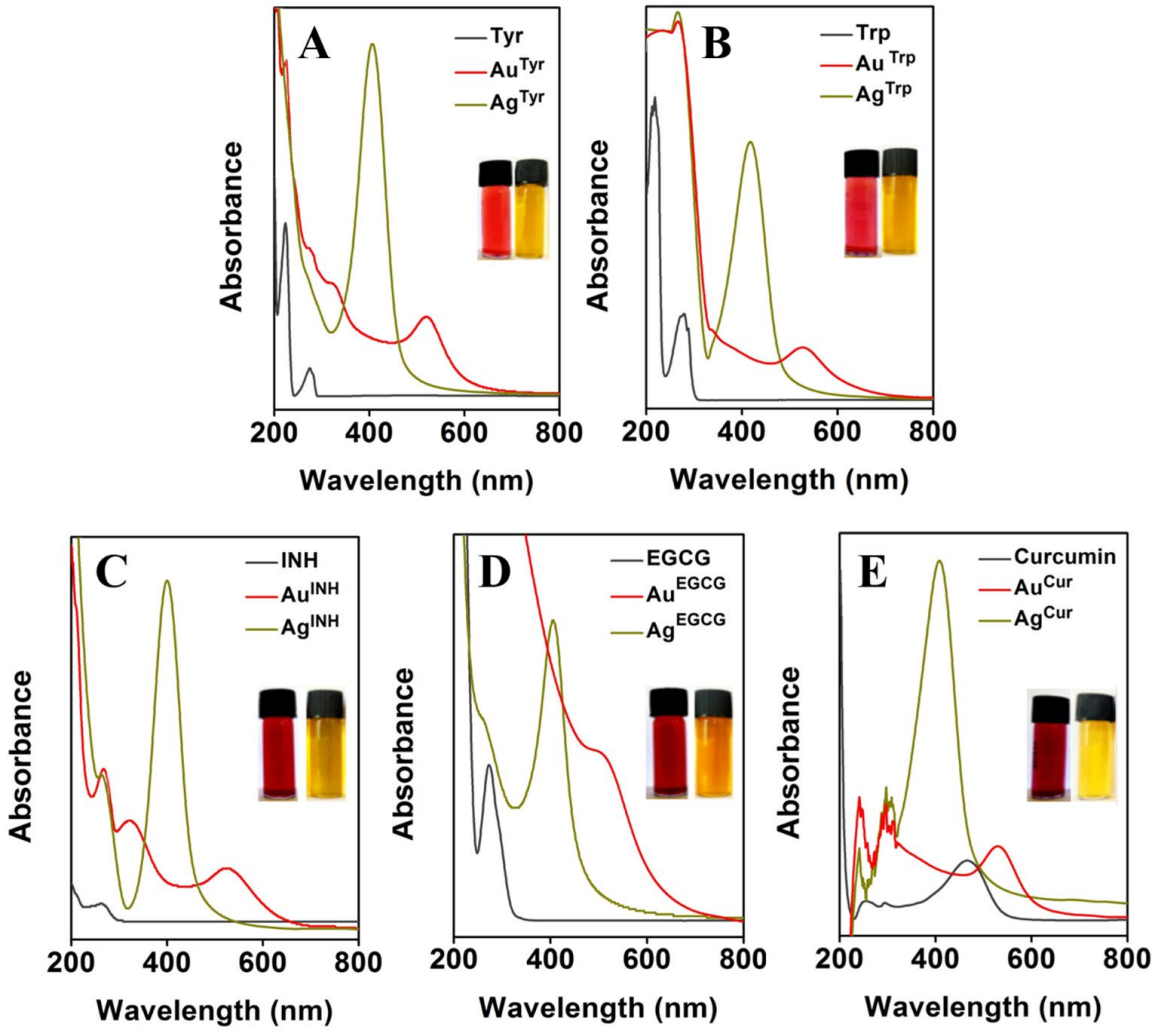
SPR absorbance spectra of Au and Ag nanoparticles. (**A**–**E**) show spectra of Au and Ag nanoparticles synthesized using Tyr, Trp, INH, EGCG, and Cur, respectively. The absorbance spectra of the pristine reducing agents are also included in the respective panels. The insets show photographs of Au (wine red color) and Ag (yellow color) nanoparticles in solution.

Previously, it has been reported that in the case of metal nanoparticles, specific solution color can advocate the formation of nanoparticles, and it can be confirmed using UV–visible spectroscopy. The development of red or wine-red color in the case of Au nanoparticles and yellow color for Ag nanoparticles solutions is characteristic of the formation of nanoparticles^18,26^. Figure 2 represents the UV–visible absorbance spectra and digital photographs of Au and Ag nanoparticles developed by employing Tyr, Trp, INH, EGCG, and Cur as reducing as well as stabilizing agents, respectively. The inset digital photographs (Fig. 2A–E) are showing the red to wine-red color or yellow color for Au and Ag nanoparticles solutions, correspondingly. The UV–Visible spectroscopy is a fundamental technique to confirm the formation of stable metal nanoparticles in an aqueous medium. In general, metal nanoparticles exhibit a unique phenomenon known as surface plasmon resonance (SPR), where the conducting electrons of metals oscillate together in resonance with certain wavelengths upon interaction with an electromagnetic field^49,50^. Therefore, UV–visible spectroscopic analysis of Au and Ag nanoparticles was carried out that confirmed the formation of nanoparticles. [AuCl_4_]^−^ and Ag^+^ ions are reduced using different reducing agents under the alkaline condition to form Tyr, Trp, INH, EGCG, and Cur-capped Au and Ag nanoparticles. Different Au nanoparticles, Au^Tyr^, Au^Trp^, Au^INH^, Au^EGCG^, and Au^Cur^, exhibited their respective SPR band maxima at ca. 520 nm (Fig. 2A), 528 nm (Fig. 2B), 532 nm (Fig. 2C), 503 nm (Fig. 2D), and 531 nm (Fig. 2E), respectively. All these SPR band maxima values are the characteristic feature of Au nanoparticles that gives rise to an absorption band at 510–540 nm. Similarly, UV–visible spectral analysis confirmed the formation of Ag nanoparticles. Ag^Tyr^, Ag^Trp^, Ag^INH^, Ag^EGCG^ and Ag^Cur^ nanoparticles display their absorption maxima at ca. 406 nm (Fig. 2A), 417 nm (Fig. 2B), 395 nm (Fig. 2C), 406 nm (Fig. 2D) and 412 nm (Fig. 2E), respectively, the distinctive feature of Ag nanoparticles^18,27,28,30^.

Moreover, a single SPR absorption maximum was detected in the spectra of Au and Ag nanoparticles, indicating that these nanoparticles may be spherical in shape without forming any other shaped structures^50–52^. It was further confirmed by microscopic imaging of nanoparticles solutions as represented in Fig. S1. The TEM micrographs of Au and Ag nanoparticles solutions confirm the spherical to quasi-spherical shape and nano-size of all the nanoparticles. In addition to being predominantly spherical or quasi-spherical shapes, some of the nanoparticles have irregular morphology, as seen in Fig. S1. Further, the size distribution profile of synthesized Au and Ag nanoparticles was determined using a DLS particle size analyzer. The DLS measurements display that all the nanoparticles are falling in the nanoscale range and are found to be sub 100 nm in size (Table 1). Additionally, the TEM micrographs of Au and Ag nanoparticles revealed that although there was no visible aggregation in the solution, the inter-particles distances were minimal. However, all these Au and Ag nanoparticles were found to be stable on storage under standard laboratory conditions in deionized water for over one and a half years. This stability is contributed due to the presence of a reducing agent (Tyr, Trp, INH, EGCG, or Cur) on the surface of nanoparticles, which also works as a stabilizing agent. Nevertheless, the fact was further confirmed by employing the zeta (ζ) potential analyzer. The ζ potential measurements offer direct evidence about the aggregation state, stability, and surface charge of the colloidal suspensions. The ζ potential values revealed that all the nanoparticles carried a negative surface charge. For Au^Tyr^, Au^Trp^, Au^INH^, Au^EGCG^, Au^Cur^, Ag^Tyr^, Ag^Trp^, Ag^INH^, Ag^EGCG^ and Ag^Cur^ nanoparticles, the ζ potential values were found to be − 12.80, − 27.63, − 17.96, − 32.40, − 24.50, − 41.10, − 33.76, − 39.83, − 27.56, and − 7.48 mV, respectively. In the case of Au^Tyr^ and Ag^Cur^ nanoparticles, it is observed that these nanoparticles bear moderate surface charge, and still, they are stable. This stability is due to the presence of Tyr and Cur on the surface of the nanoparticles; they stabilize the Au^Tyr^ and Ag^Cur^ nanoparticles by forming a protective steric shield around the nanoparticles^45,53,54^. Additionally, it has been reported that the negative repulsion forces in the solution help in high stability and high dispersity of the nanoparticles. The high ζ potential values for all nanoparticles were further confirmed by the stability of Au and Ag nanoparticles prepared using different reducing agents.

**Table 1:** Average particles size (hydrodynamic radius) and zeta potential (**ζ**) measured by DLS and zeta potential analyzer of Au and Ag nanoparticles prepared using Tyr, Trp, INH, EGCG and Cur.

Sample	Average hydrodynamic radii (nm)	ζ potential (mV)
Au^Tyr^ nanoparticles	58.26 ± 0.68	− 12.80 ± 0.34
Au^Trp^ nanoparticles	21.60 ± 0.96	− 27.63 ± 0.65
Au^INH^ nanoparticles	98.86 ± 1.32	− 17.96 ± 0.49
Au^EGCG^ nanoparticles	41.39 ± 1.47	− 32.40 ± 0.81
Au^Cur^ nanoparticles	56.80 ± 0.83	− 24.50 ± 0.27
Ag^Tyr^ nanoparticles	38.14 ± 0.93	− 41.10 ± 0.18
Ag^Trp^ nanoparticles	50.04 ± 0.81	− 33.76 ± 0.54
Ag^INH^ nanoparticles	11.65 ± 1.12	− 39.83 ± 0.45
Ag^EGCG^ nanoparticles	33.14 ± 0.72	− 27.56 ± 0.39
Ag^Cur^ nanoparticles	83.22 ± 0.69	− 07.48 ± 0.63

We next employed FTIR spectroscopy to identify the coordination of coated nanoparticles, and also to obtain direct evidence for the presence of functional groups in the surface corona. The distinguishing functional group vibrational frequencies of reducing molecules (Tyr, Trp, INH, EGCG, or Cur) on the surface of Au and Ag nanoparticles confirmed their presence (Fig. S2). For example, Au^INH^ nanoparticles reveal characteristic INH functional vibrational frequencies at 3277 cm^−1^ and 1634 cm^−1^ for N–H stretching and carbonyl group C=O, whereas these were shown at 3263 cm^−1^ and 1634 cm^−1^ for Ag^INH^ nanoparticles. Similarly, the FTIR spectra of Trp synthesized Au^Trp^, and Ag^Trp^ nanoparticles show the carbonyl stretching frequency of oxidised-Trp at 1636 cm^−1^ and 1635 cm^−1^, respectively. These spectral frequencies are attributed to the formation of carboxylate ions during reaction^55^. Likewise, Tyr capped-Au^Tyr^ and Ag^Tyr^ nanoparticles have a carbonyl stretching frequency around 1639 cm^−1^ and 1635 cm^−1^, due to the carboxylate ion formation. The observed vibrational frequencies in all the cases are concomitant to the reported distinguishing functional group vibrational frequencies^26,40,45^. The presence of functional groups vibrational frequencies even after Au and Ag nanoparticles formation with changes in the wavenumber verified the existence of oxidized-reducing moieties on the surface of respective nanoparticles, as illustrated in Fig. 1D.

It is apparent from the above studies that the nanoparticles prepared with Tyr, Trp, INH, EGCG, or Cur produced a biomolecular corona supported on inorganic metal (Au and Ag). This observation led to our hypothesis that nanoparticles may possess specific biological effects. Therefore, we assayed for biological activities and cytotoxic properties of the nanoparticles.

**Figure 3:**
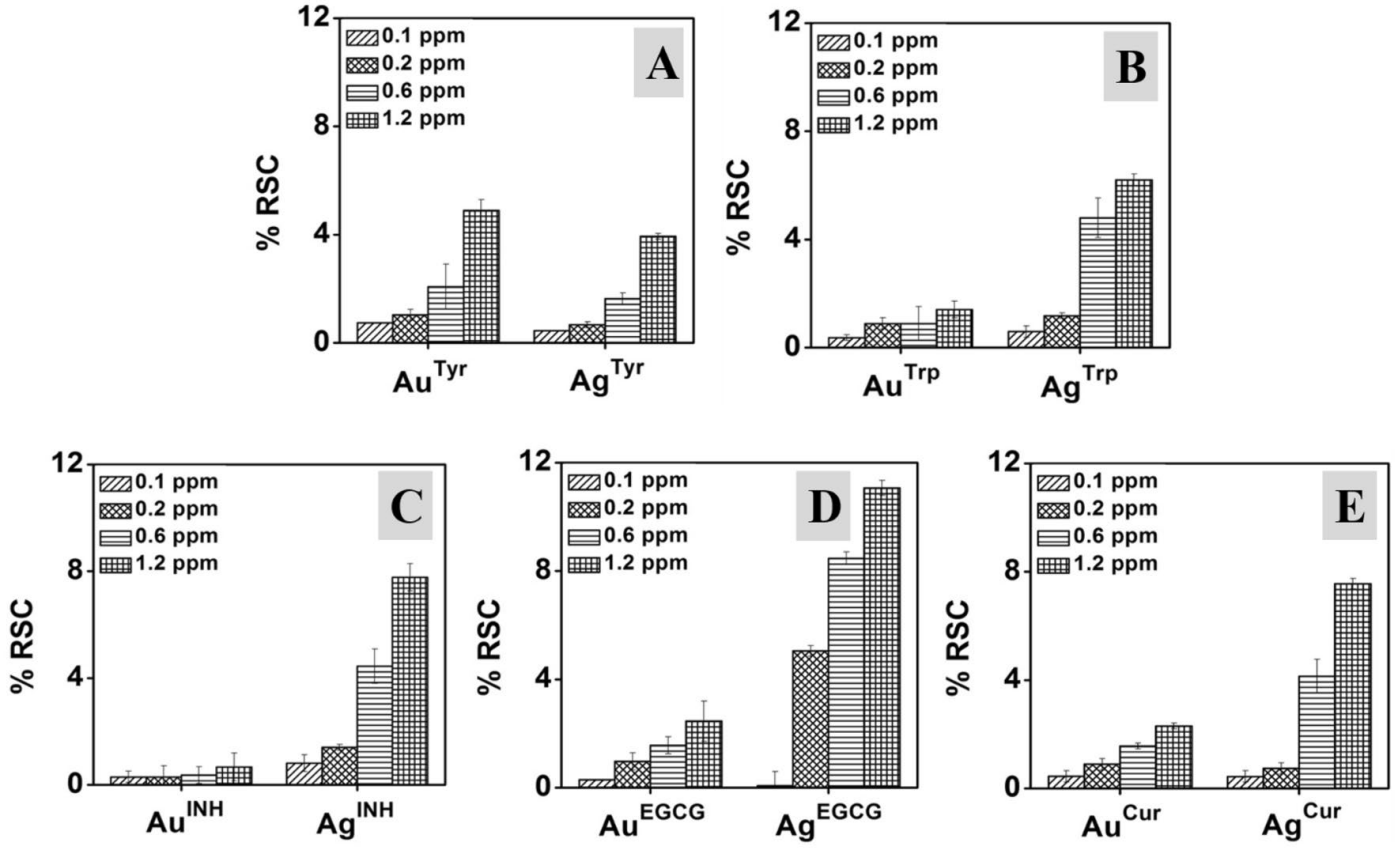
Radical scavenging capacity (RSC) of Au and Ag nanoparticles evaluated using ABTS. (**A**–**E**) show the % RSC of Au and Ag nanoparticles synthesized with Tyr, Trp, INH, EGCG, and Cur, respectively.

It has been reported that aging, cancer, cataracts, brain dysfunction, and cardiovascular diseases are associated with an excess amount of reactive oxygen species (ROS) in the body. The excess production of ROS influences metabolic processes and plays an essential role in the development of diseases^56^. On the contrary, nanoparticles have the competency to scavenge the free radicals, and they can play an important role in preventing various diseases linked with the free radicals attack on cells and biological targets^57,58^. In this context, various nanoparticles, such as silica nanoparticles, iron-oxide nanoparticles, cerium oxide nanoparticles, metal nanoparticles, and organic (i.e., melanin, lignin) nanoparticles have been designed and assessed for their potential free radical scavenging capacity (RSC)^18,30,59–62^. Here, we have investigated the in vitro RSC of different Au and Ag nanoparticles employing ABTS assay. In this test, the decolorization of ABTS radical cation (ABTS^·+^) solution in the presence of nanoparticles suggestive of the in vitro RSC of the respective nanoparticle. As illustrated in Fig. 3A–E, the RSC of nanoparticles was dose-dependent, and with the increasing doses of nanoparticles, the RSC of both Au and Ag nanoparticles for all the ten nanoparticle systems increased till the evaluated doses. For example, Au^Trp^ nanoparticles show 0.37%, 0.89%, 0.89% and 1.41% of RSC, and Ag^Trp^ nanoparticles display 0.59%, 1.18%, 4.80% and 6.02% of RSC at 0.1, 0.2, 0.6 and 1.2 ppm dosages, respectively (Fig. 3B). Moreover, with the increasing doses, the differences in %RSC becomes notable, and a similar trend is observed in all the cases. However, the impact on the RSC varied in the case of different nanoparticles, which can be attributed to the alteration in the biomolecular surfaces and metal content (Au or Ag) of nanoparticles. For example, at a constant metal concentration of 1.2 ppm (Au and Ag), it was found that the Au^Tyr^, Au^Trp^, Au^INH^, Au^EGCG^ and Au^Cur^ nanoparticles display 4.88%, 1.41%, 0.67%, 2.46% and 2.31% RSC, whereas, equivalent Ag^Tyr^, Ag^Trp^, Ag^INH^, Ag^EGCG^ and Ag^Cur^ nanoparticles display 3.93%, 6.20%, 7.78%, 11.06% and 7.56% RSC, respectively (Fig. 3). In the case of Au nanoparticles, the in vitro RSC action is shown as Tyr ˃ EGCG ˃ Cur ˃ Trp ˃ INH, whereas for Ag nanoparticles, the RSC action is revealed as EGCG ˃ INH ˃ Cur ˃ Trp ˃ Tyr at 1.2 ppm metal fraction. It is interesting that the metal fraction is constant in all cases. However, differential activity is observed, which indicates that the RSC of nanoparticles can be attributed to the bioactive surfaces of individual nanoparticles. Nevertheless, observations of Fig. 3 also reveal that Ag nanoparticles show higher RSC compare to Au nanoparticles, which is complimentary to reported literature^30^. As discussed, at the constant 1.2 ppm dosages of Au^Trp^ and Ag^Trp^ nanoparticles, 1.41% and 6.20% RSC were observed, respectively. This further specifies that in addition to distinctive biomolecular surfaces, the metal core also plays an important role in determining the in vitro RSC competency of nanoparticles. For control experiments, and to confirm the role of the metallic core in RSC of nanoparticles^18^, the pristine reducing agents and precursor metal ions were also employed to validate the intrinsic nature of metal core in RSC of nanoparticles (Fig. S3).

**Figure 4:**
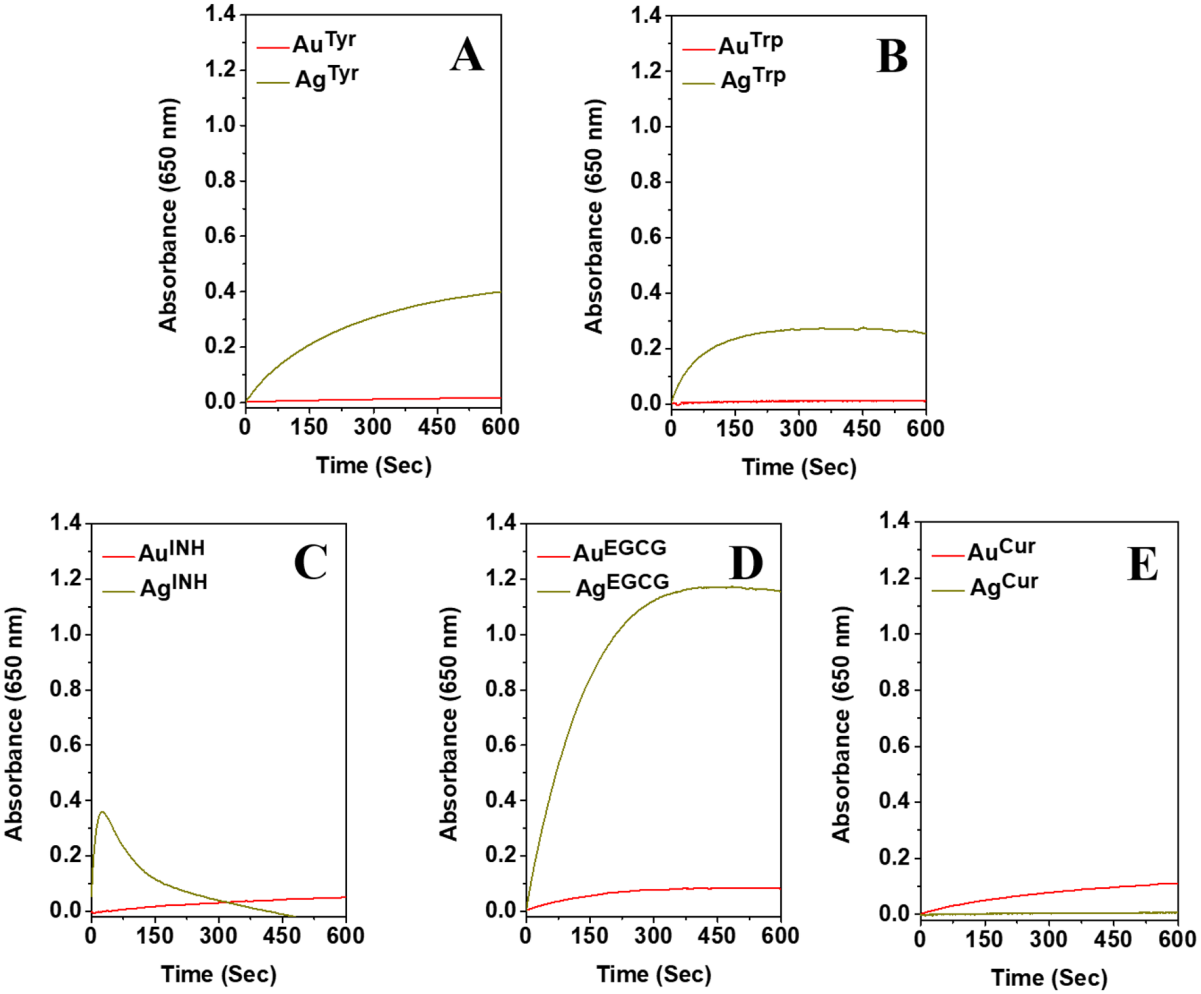
Peroxidase mimicking activity of Au and Ag nanoparticles synthesized with Tyr, Trp, INH, EGCG, and Cur, respectively. The concentration of Au or Ag is 1.2 ppm.

It is further envisioned that due to the presence of bioactive surfaces on nanoparticles, the nanoparticles may exhibit nanozyme-mimic activity. It was confirmed employing TMB, which is a chromogenic substrate and, in general, used to evaluate the peroxidase-like activity of nanomaterials^18,26,30^. The oxidation of chromogenic TMB in the presence of H_2_O_2_ can be catalyzed by metal nanoparticles, and it can be observed from the blue coloration of the reaction mixture. It has been reported that the Ag nanoparticles have higher peroxidase-like activity than Au nanoparticles^18,26^. Here, we report a similar trend for Tyr, Trp, INH and EGCG synthesized nanoparticles, as shown in Fig. 4 (for 1.2 ppm metal concentration) and Fig. S4 (for 0.6 ppm metal concentration). The observations support the reported literature and validate the role of the metal fraction of nanoparticles in their peroxidase nanozyme-mimic activity. However, it was interesting to detect that in Cur reduced nanoparticles at a given metal concentration (1.2 ppm, Fig. 4), Au^Cur^ has higher peroxidase-mimic activity compare to Ag^Cur^ nanoparticles. This observation indicates that in addition to the metal core of the individual nanoparticles, the bioactive surfaces of nanoparticles can also be influential in determining their behavior. In addition, when we compared the activity of different biomolecular surface’s reliant nanozyme-like activity, it was revealed that for various Ag nanoparticles, the activity was changed, and it was EGCG ˃ Tyr ˃ INH ˃ Trp ˃ Cur, respectively. In this context, it has also been reported that the size of the nanozyme is important, and smaller nanoparticles show higher enzyme-like activity due to the high surface-to-volume ratio^63,64^. In one of the reports, a size‐dependent catalytic activity of core‐shell nanoparticles is also discussed, and the particles were found to have biosensing applications^65^. Additionally, the influential role of Au and Ag nanoparticles surface corona, and metallic core reliant RSC and in-vitro peroxidase enzyme-like behavior has been confirmed^30^. Therefore, the active role of the surface of the nanoparticles in determining their behavior must be assigned carefully, and all the other parameters must be controlled.

**Figure 5:**
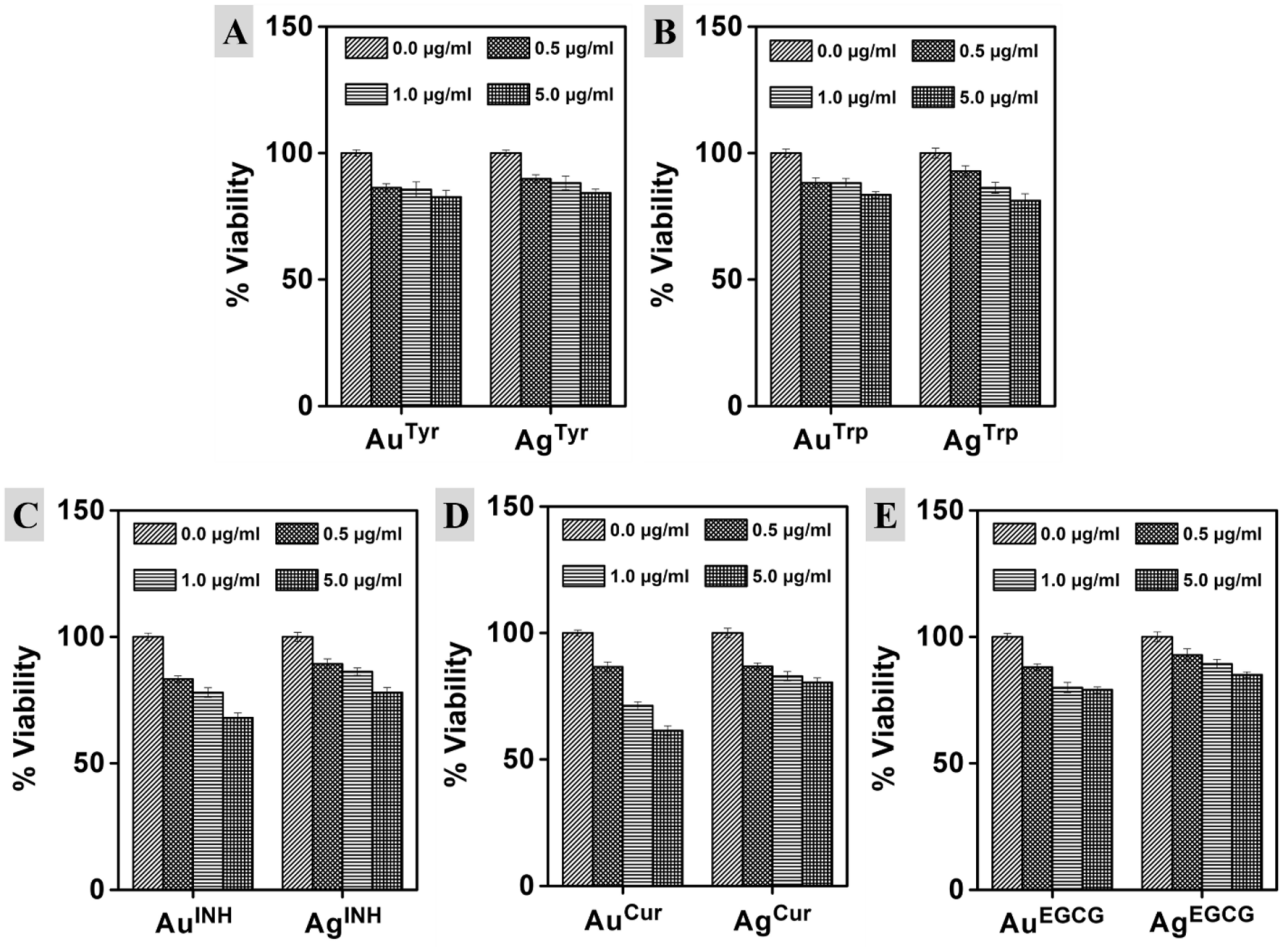
Effect of Au and Ag nanoparticles on the viability of m5S mouse skin fibroblasts. 0.5, 1.0 and 5.0 µg/ml represent metal content in the respective nanoparticle’s solution. The 0.0 µg/ml is the control solution consisting of milliQ water without any nanoparticles. Data represent mean ± SD (n = 3).

**Figure 6:**
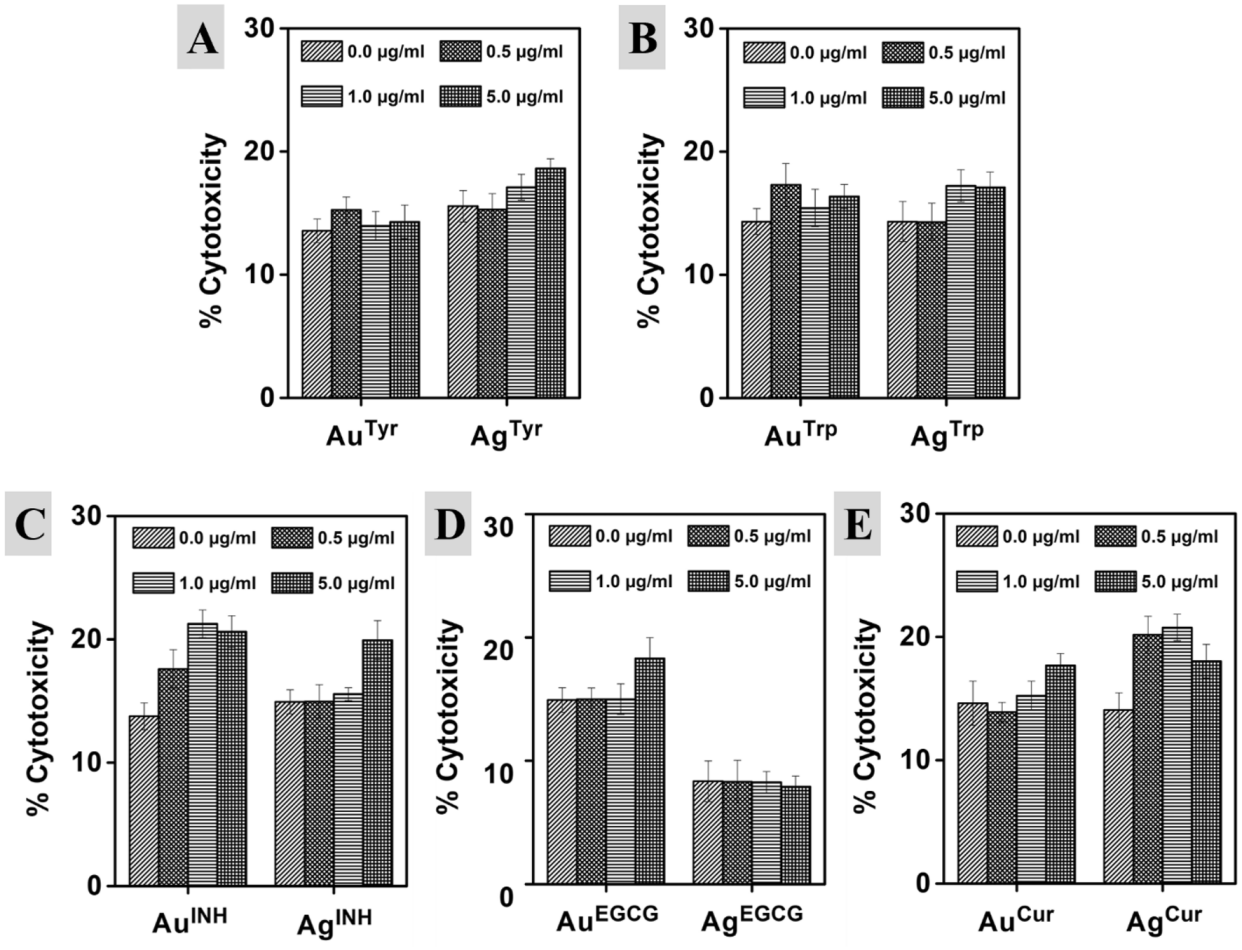
Cytotoxicity profile of Au and Ag nanoparticles synthesized with Tyr, Trp, INH, EGCG, and Cur, respectively. Cytotoxicity is taken as % increase in LDH release from m5S skin fibroblast cells after exposure to nanoparticles. 0.5, 1.0 and 5.0 µg/ml represent metal content in the respective solutions. 0.0 µg/ml is the control solution containing milliQ water without any nanoparticles. Data represent mean ± SD (n = 3).

After in vitro assessment, to verify the impact of metal nanoparticles in conjugation with bioactive surfaces on cell viability/cytotoxicity, we employed them against m5S mouse skin fibroblasts. The multiplex viability/cytotoxicity assay kit was used to determine the cell viability and cytotoxicity. The multiplex viability/cytotoxicity assay kit comprises cell counting kit-8 (CCK-8) along with the LDH examination kit. In this, the CCK-8 can measure the dehydrogenases activity (in living cells) by determining the number of viable cells, whereas the released LDH from damaged cell membranes can be determined by employing the LDH assay kit to confirm the number of dead cells (cytotoxicity). In a single point incubation period of 16 h with various doses of nanoparticles, there was a reduction in the viability of cells with the increasing doses, as illustrated in Fig. 5. Amino acid synthesized Au nanoparticles showed almost similar behavior, and there was no significant difference even at different concentrations. For example, Au^Tyr^ nanoparticles showed 86.27%, 85.60% and 82.63% cell viability at 0.5, 1.0 and 5.0 µg/ml treatments, whereas, Au^Trp^ nanoparticles showed 89.87%, 88.23% and 84.27% cell viability respectively. A similar trend was observed for Ag^Tyr^ nanoparticles, whereas in the case of Ag^Trp^ nanoparticles, 92.8–81.27% cell viability was detected at 0.5 and 5.0 µg/ml, respectively. Although the higher impact on cell viability was reported in the case of Ag^Trp^ with the increasing doses at a specific concentration, there was no significant difference than other nanoparticles prepared using amino acids. For examples, at a constant 5.0 µg/ml concentration Au^Tyr^, Au^Trp^, Ag^Tyr^ and Ag^Trp^ nanoparticles reveal 82.63%, 83.57%, 84.27% and 81.27% cell viability respectively. On the other hand, in the case of other reducing agents, differential action has been reported. For instance, at the constant 5.0 µg/ml concentration Au^Tyr^, Au^Trp^, Au^INH^, Au^EGCG^, and Au^Cur^ nanoparticles show 82.63%, 83.57%, 68.10%, 79.10% and 61.37% cell viability respectively. The differential cell viability of similar core containing nanoparticles with a constant concentration reveals that the biomolecule surface of the nanoparticles is an essential property of any material to determine their biological capabilities. Similarly, there was no significant difference in the case of an amino acid (Tyr and Trp) synthesized nanoparticles to confirm the role of the metal core. However, for all the other reducing agents, the impact of the metal core was noticeable, confirming the importance of nanoparticle composition. For a case, Au^Cur^ and Ag^Cur^ nanoparticles show 61.37% and 80.50% cell viability, respectively. It indicates that the metal fraction is also an important factor in controlling the biological behavior of nanoparticles since Cur surfaces are constant on both the nanoparticles. A similar trend was observed for INH and EGCG reduced nanoparticles of Au and Ag. Subsequently, a decrease in the viable cell number may be the hallmark of cytotoxicity originating from nanoparticles. Therefore, in the present study, we have further performed a dose-dependent cell cytotoxicity assessment of Au and Ag nanoparticles. Furthermore, it is important to state that the cell cytotoxicity may be dependent on the surface moieties in addition to the metal fraction of nanoparticles, as discussed above for cell viability. The cell cytotoxicity was complimentary to the cell viability analysis of various nanoparticles, as illustrated in Fig. 6. The cell viability and cytotoxicity in similar experimental conditions were also determined for all the pristine reducing agents (Tyr, Trp, INH, EGCG, and Cur) and free metal ions (AuCl_4_^−^ and Ag^+^ ions) as illustrated in Fig. S5. Furthermore, to validate the hypothesis, we have employed various Au and Ag nanoparticles against macrophages, which are the master cells of the innate immune system, and contribute to the immunity, repair, and tissue hemostasis (Fig. S6). The Tyr, EGCG, and Cur synthesized nanoparticles stimulated cells, and there was an increment in the cell numbers at 1.0 and 5.0 µg/ml. In the recent past, comparable observations have been reported in quercetin capped metal nanoparticles^18^. Here, the observations complement that the cell viability of the nanoparticles can be controlled by a suitable surface corona of the particles in addition to the metallic core of Au or Ag nanoparticles.

**Figure 7:**
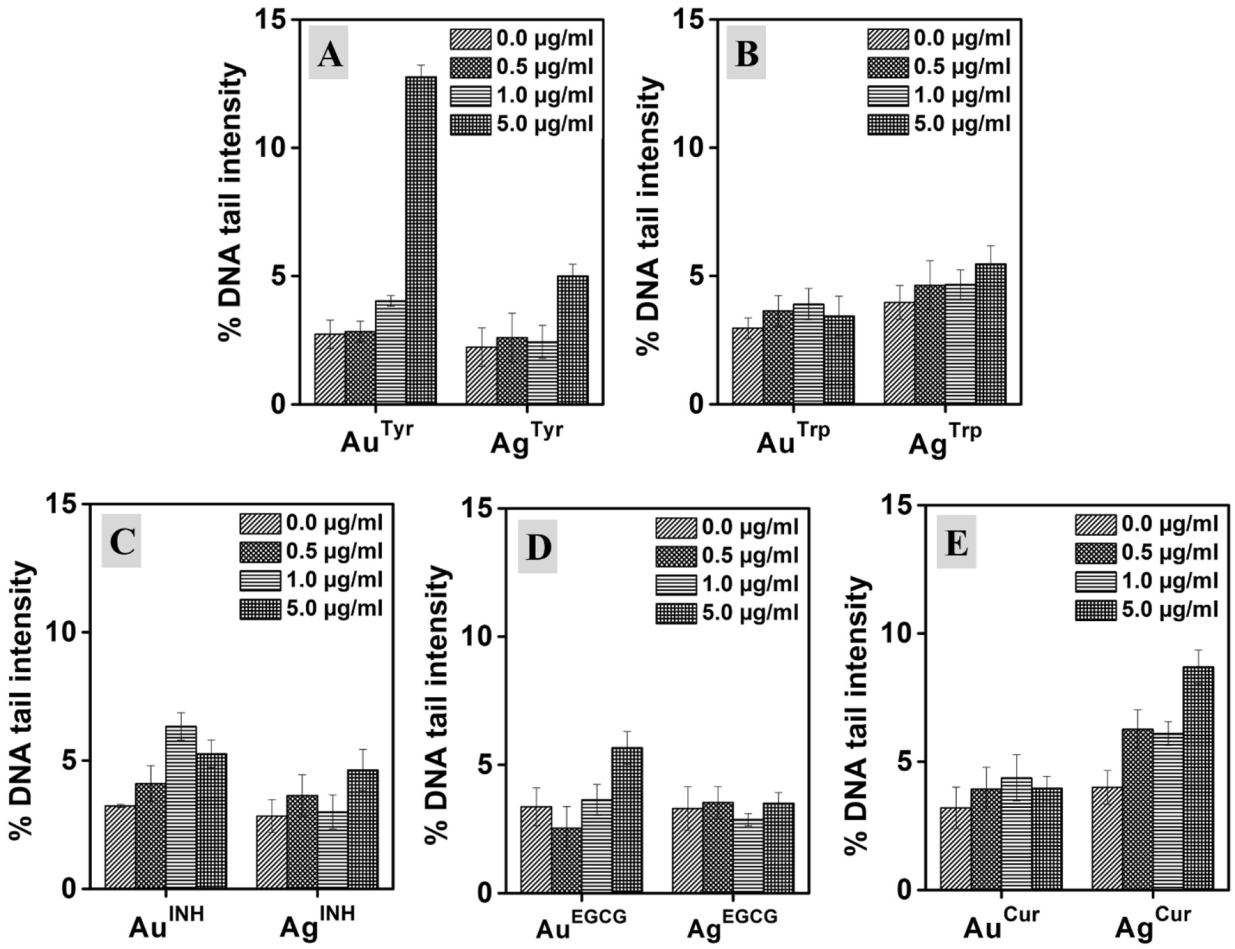
Genotoxicity (expressed as %DNA tail intensity) induced in m5S mouse skin fibroblast cells following 24 h of exposure to Au and Ag nanoparticles synthesized with Tyr, Trp, INH, EGCG, and Cur, respectively. 0.5, 1.0 and 5.0 µg/ml represent metal content in the respective nanoparticle’s solution. 0.0 µg/ml is the control which contained milliQ water but without any nanoparticles.

Nevertheless, many metal and metal oxide nanoparticles have been reported for their potential toxicity, but the genotoxicity of nanomaterials is relatively unexplored, and inconsistencies are found in the literature^66,67^. However, urgent attention is required towards the evaluation of potential genotoxic properties of metallic nanoparticles, which can be crucial in regulatory safety assessment. Therefore, we have evaluated the nanoparticles-induced genotoxicity by employing single-cell DNA damage assay, and results are expressed in % DNA tail intensity with respect to treatments in Fig. 7. Here, different Au and Ag nanoparticles of various concentrations (0.5, 1.0, and 5.0 µg/ml) were incubated with m5S mouse skin fibroblasts for 24 h, followed by cell harvesting and DNA damage assessment using Comet Analyzing Software. As illustrated in Fig. 7A, in terms of the extent of magnitude, only Au^Tyr^ of 5.0 µg/ml treatment induced greater DNA damage with around four-fold tail intensification compared to control. However, it was surprising to witness that all the Au and Ag nanoparticles synthesized with different reducing agents exhibited lower levels of DNA damage as measured by tail intensity. Moreover, it is interesting to observe that the nanoparticles of Au and Ag showed reduced genotoxicity when compared with the corresponding free metal ions (Fig. S7). Free metal ions are known to have a higher level of genotoxicity originating from various chemical mechanisms^68^. Furthermore, in the case of pristine INH, the antibiotic was found to induce DNA damage. However, when it is present on the surface of nanoparticles (Au^INH^ and Ag^INH^), the toxicity impact was controlled. It is interesting that in the free state, both the ions and INH showed significant damage to DNA (Fig. S7), but in the form of Au^INH^ and Ag^INH^ nanoparticles, the toxic effect was deactivated as illustrated in Fig. 7C. These results are concomitant to investigated cell viability and cytotoxicity assessments, as discussed above. However, to further confirm the results of genotoxicity (DNA tail assay), the DNA ladder (DNA fragmentation) assessment was performed.

**Figure 8:**
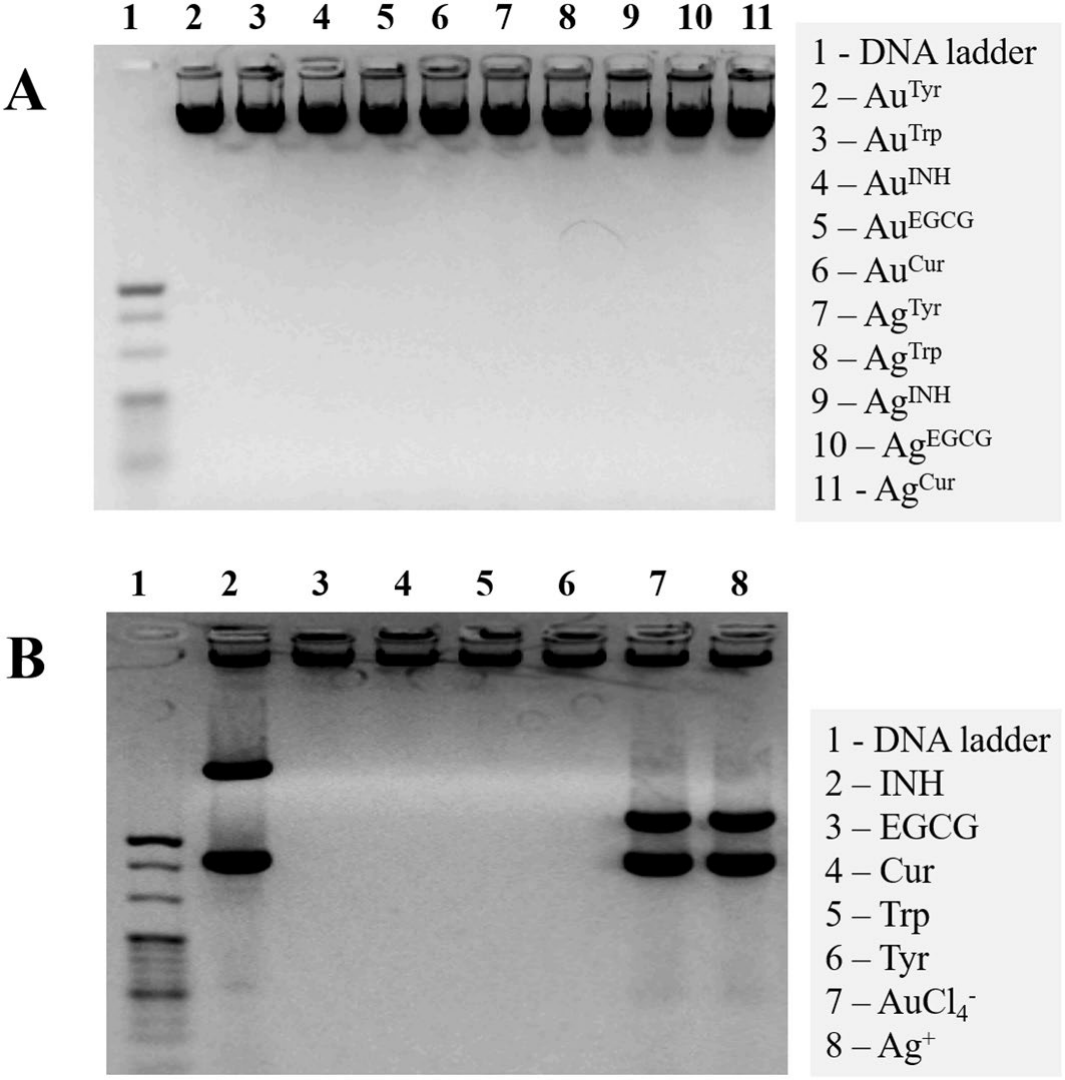
Induction of apoptosis by exposure to Au and Ag nanoparticles: (**A**) DNA fragmentation profiling in m5S skin fibroblast cells treated with different Au and Ag nanoparticles synthesized with Tyr, Trp, INH, EGCG, and Cur, respectively. (**B**) Response to pristine reducing agents with free metal ions used as in controls. Lane 1 shows a standard molecular size marker (1 Kb).

The DNA ladder assay is an essential method for the rapid screening of DNA break in cell populations. The specific ladder pattern is an indication of apoptosis by the activation of a nuclear endonuclease^69,70^. In this technique, DNA cleavage can be seen in the form of a ladder pattern due to damage caused by nanoparticles or by the activation of the nuclear endonuclease. Moreover, DNA fragments after treatment with nanoparticles can be visualized in standard agarose gel electrophoresis. Therefore, DNA ladder assay was performed. As illustrated in Fig. 8A, it was confirmed that various Au and Ag nanoparticles do not induce apoptosis in m5S skin fibroblasts. It was interesting to observe that the DNA was damaged by pristine INH (Fig. 8B, Lane 2) and free metal ions (Fig. 8B, Lane 7, and 8). However, when the INH was used as a reducing agent, it did not promote any fragmentation to DNA after treatment with Au and Ag nanoparticles, as shown in Fig. 8A, Lane 4, and 9, respectively. This observation specifies that toxicity induced by DNA fragmentation must be considered while it is being assigned to nanoparticles. The individual surface moieties and free metal ions can show DNA fragmentation, leading to apoptosis. However, this nature may be altered during the synthesis of nanoparticles, and carefully designed nanoparticles can behave differently, as shown in the case of INH-reduced Au^INH^ and Ag^INH^ (Fig. 8). On the contrary, other reducing agents, namely Tyr, Trp, EGCG, and Cur were found to have no apoptosis activity (Fig. 8B, Lane 3–6), and when they are used as reducing agents, the respective nanoparticles (both Au and Ag) were also inheriting the same nature. It is an interesting observation that during the nanoparticles synthesis, the surface moieties controlled the inherent nature of nanoparticles, and the characteristic metal ions nature was altered in the nanoparticulate form as illustrated in Fig. 8A,B. From the discussion, it can be concluded that the biological action of nanoparticles is derived from the complementary effect of the metallic core and biomolecular surfaces of nanoparticles. However, it is imperative to state that all the nanoparticles formulated in this study have a distinctive hydrodynamic radius, which may also contribute to their biological interactions in addition to their composition and surfaces. Therefore, we must be cautious while assigning the biological characteristic of any physicochemical property of nanoparticles.

## Conclusions

In the present study, single-step approaches to prepare Au and Ag nanoparticles with a variety of surface corona have been reported (Fig. S8). We have achieved different and bioactive surface corona using amino acids (Tyr and Trp), antibiotics (INH), and plant-derived polyphenol (EGCG and Cur) molecules. The impact of the metal composition of nanoparticles and their respective surface corona have been established in vitro using m5S mouse skin fibroblasts and RAW 264.2 macrophages. We have shown that surface corona dictates the physicochemical properties and the biological interactions of the Ag and Au nanoparticles. Overall, the present study suggests novel approaches towards the engineering of surface properties and the composition of nanoparticles to enhance their biological applicability. Additional studies are required to examine the interaction of nanoparticles with biological fluids for predicting their activities in vivo.

## Supplementary information

Supplementary Figure(s) 1.

## Abbreviations

α-MEM: Alpha-minimum essential medium
AAS: Atomic absorption spectroscopy
CCK: Cell counting kit
Cur: Curcumin
DLS: Dynamic light scattering
DMSO: Dimethyl sulfoxide
EDTA: Ethylenediaminetetraacetic acid
EGCG: Epigallocatechin gallate
EtBr: Ethidium bromide
FBS: Fetal bovine serum
FTIR: Fourier-transform infrared spectroscopy
INH: Isonicotinylhydrazide
PBS: Phosphate-buffered saline
PPT: Plasmonic photothermal therapy
RSC: Radical scavenging capacity
SERS: Surface-enhanced Raman scattering
SPR: Surface plasmon resonance
ROS: Reactive oxygen species
Trp: Tryptophan
Tyr: Tyrosine
