# Supplementary material for: Engineering bioactive surfaces on nanoparticles and their biological interactions
Source: Sci Rep. 2020 Nov 12;10:19713. doi: 10.1038/s41598-020-75465-z (PMC7665184; doi:10.1038/s41598-020-75465-z)
Supplement: Supplementary file 1 — Supplementary Figure(s) 1. [file 41598_2020_75465_MOESM1_ESM.docx]

**Supplementary Material**

***for***

**Engineering** **bioactive surfaces on nanoparticles and** **their biological interactions**

Meghana Matur^1^, Harishkumar Madhyastha^2^, Shruthi T.S^1^, Radha Madhyastha^2^, S. P. Srinivas^3^, Navya P N^1,4*^, Hemant Kumar Daima^1,5*^

^1^Nano-Bio interfacial research laboratory (NBIRL), Department of Biotechnology,

Siddaganga Institute of Technology, Tumkur-572103, Karnataka, India

^2^Department of Applied Physiology, Faculty of Medicine, University of Miyazaki,

Miyazaki-8891692, Miyazaki, Japan

^3^School of Optometry, Indiana University, Bloomington,

Indiana-47405, USA

^4^Department of Biotechnology, Bannari Amman Institute of Technology,

Sathyamangalam, Erode, Tamil Nadu, 638401, India

^5^Amity Center for Nanobiotechnology & Nanomedicine (ACNN), Amity Institute of Biotechnology, Amity University Rajasthan, Kant Kalwar, NH-11C,

Jaipur-Delhi Highway, Jaipur-303002, Rajasthan, India

^*^Corresponding authors – Telephone: +91 8884774863, +91 8764402136

E-mail: hkdaima@jpr.amity.edu, hkdaima@gmail.com, navyapn@sit.ac.in

ORCID (Hemant K. Daima): http://orcid.org/0000-0002-9109-430X

(Navya PN): https://orcid.org/0000-0001-9345-8253


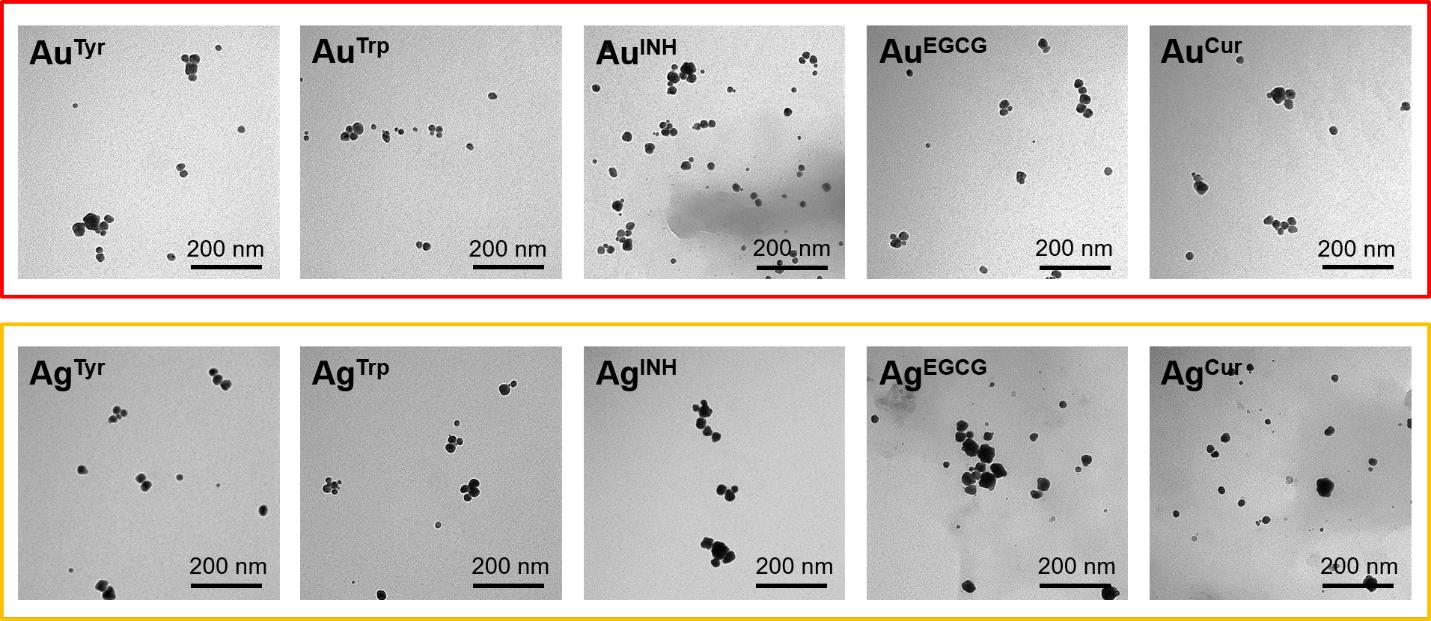


**Figure S1:** TEM micrographs of the Au and Ag nanoparticles, which were synthesized with Tyr, Trp, INH, EGCG, and Cur as reducing and stabilizing agents.


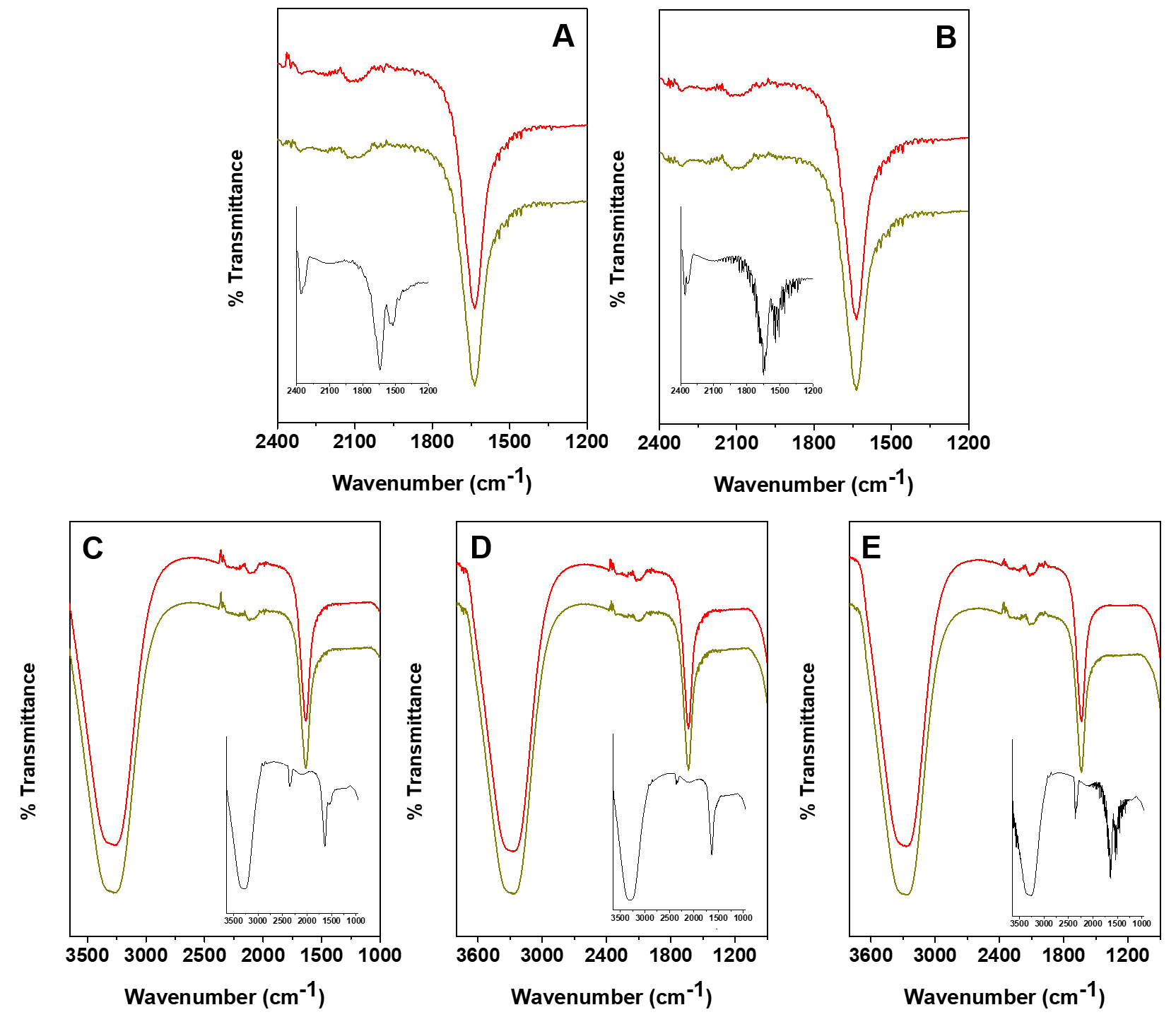


**Figure S2:** FTIR spectra of the Au and Ag nanoparticles synthesized with (A) Tyr, (B) Trp, (C) INH, (D) Cur, and (E) EGCG, respectively. The nanoparticles show distinct functional group vibrational frequencies corresponding to the different reducing agents. The inset in each of the panels indicates the spectrum of the corresponding reducing agent.

**
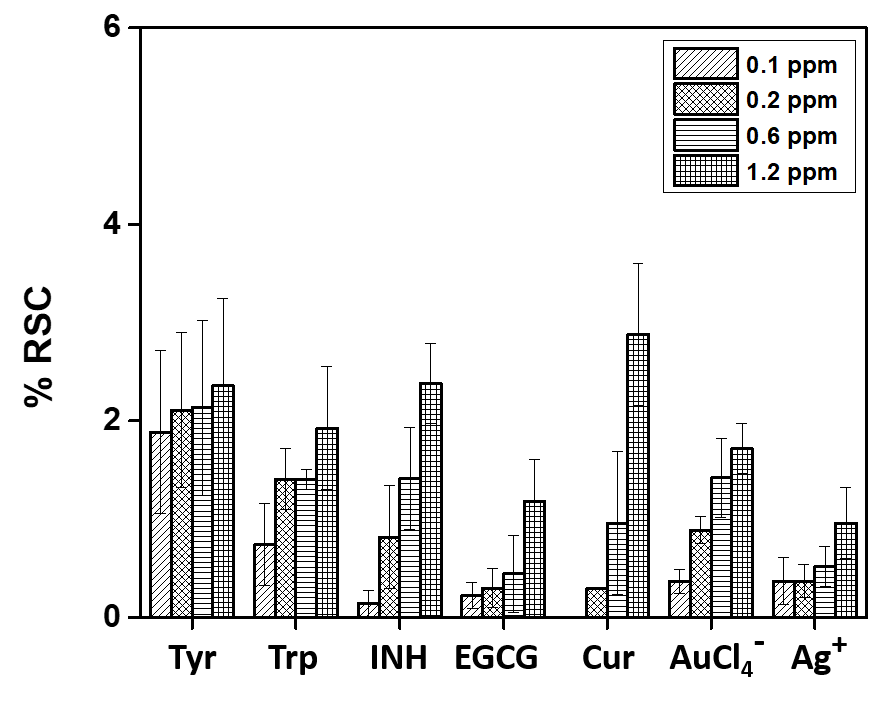
**

**Figure S3:** Assessment of %RSC of Tyr, Trp, INH, EGCG, Cur, AuCl_4_^-^ and Ag^+^, respectively.


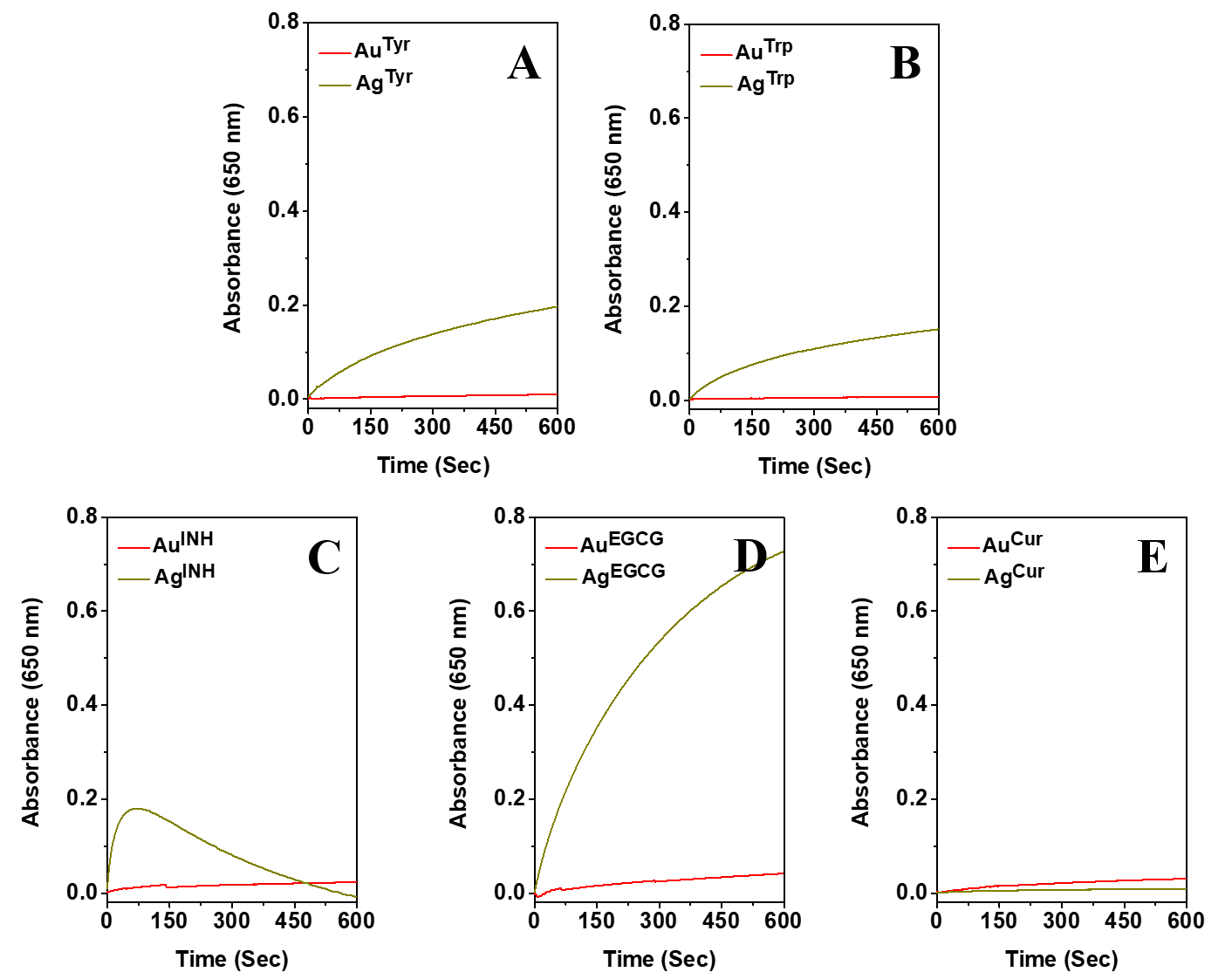


**Figure S4:** Peroxidase-mimicking activity of the Au and Ag nanoparticles synthesized with Tyr, Trp, INH, EGCG, and Cur. The metal concentration is 0.6 ppm in both Au and Ag nanoparticles.


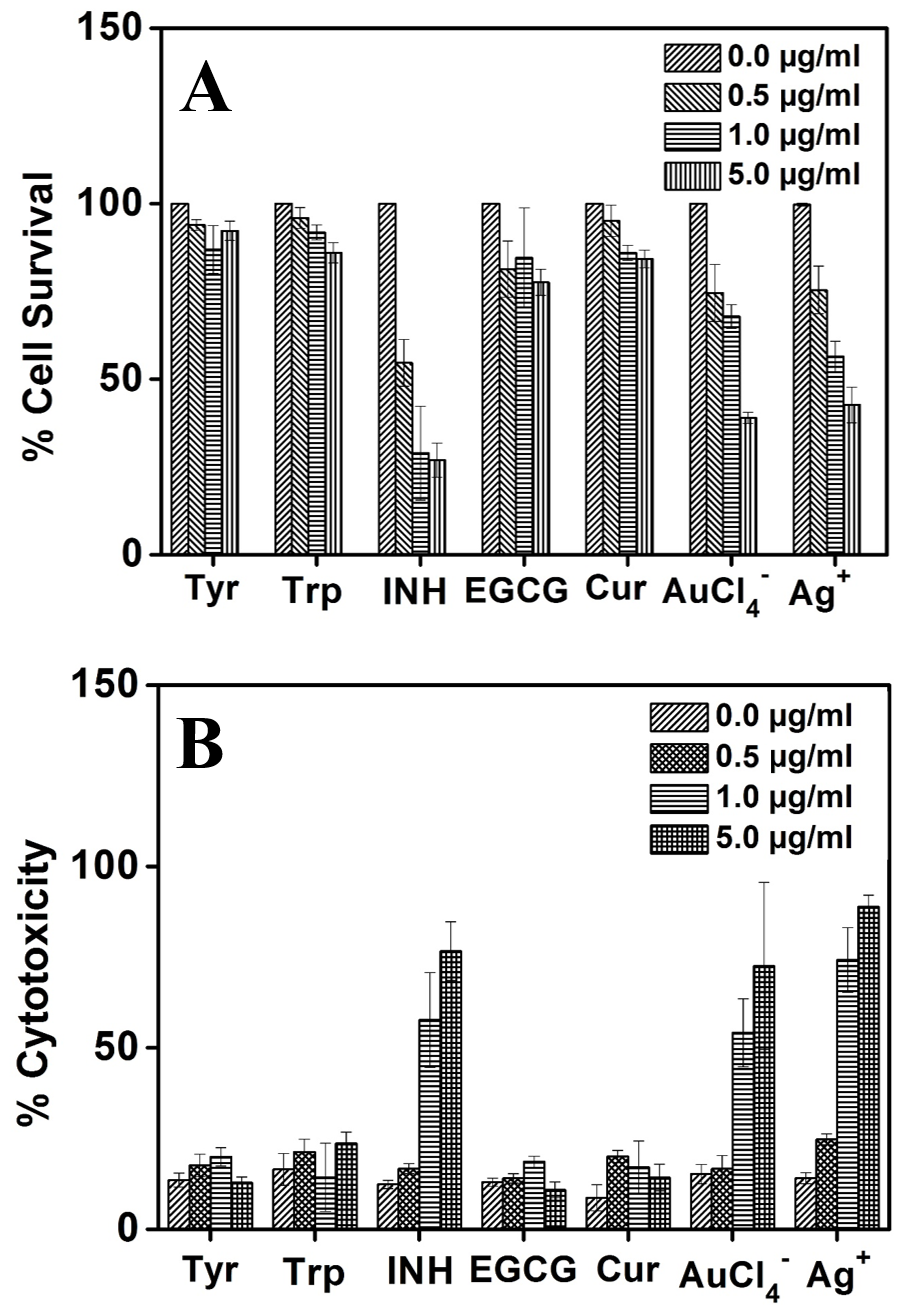


**Figure S5:** Cell viability and cytotoxicity assessment of Tyr, Trp, INH, EGCG, Cur, AuCl_4_^-^ and Ag^+^, respectively. The cell viability and cytotoxicity by treating m5S mouse skin fibroblasts with pristine reducing agents and free metal ions. Data represent mean ± SD (n=3).

**
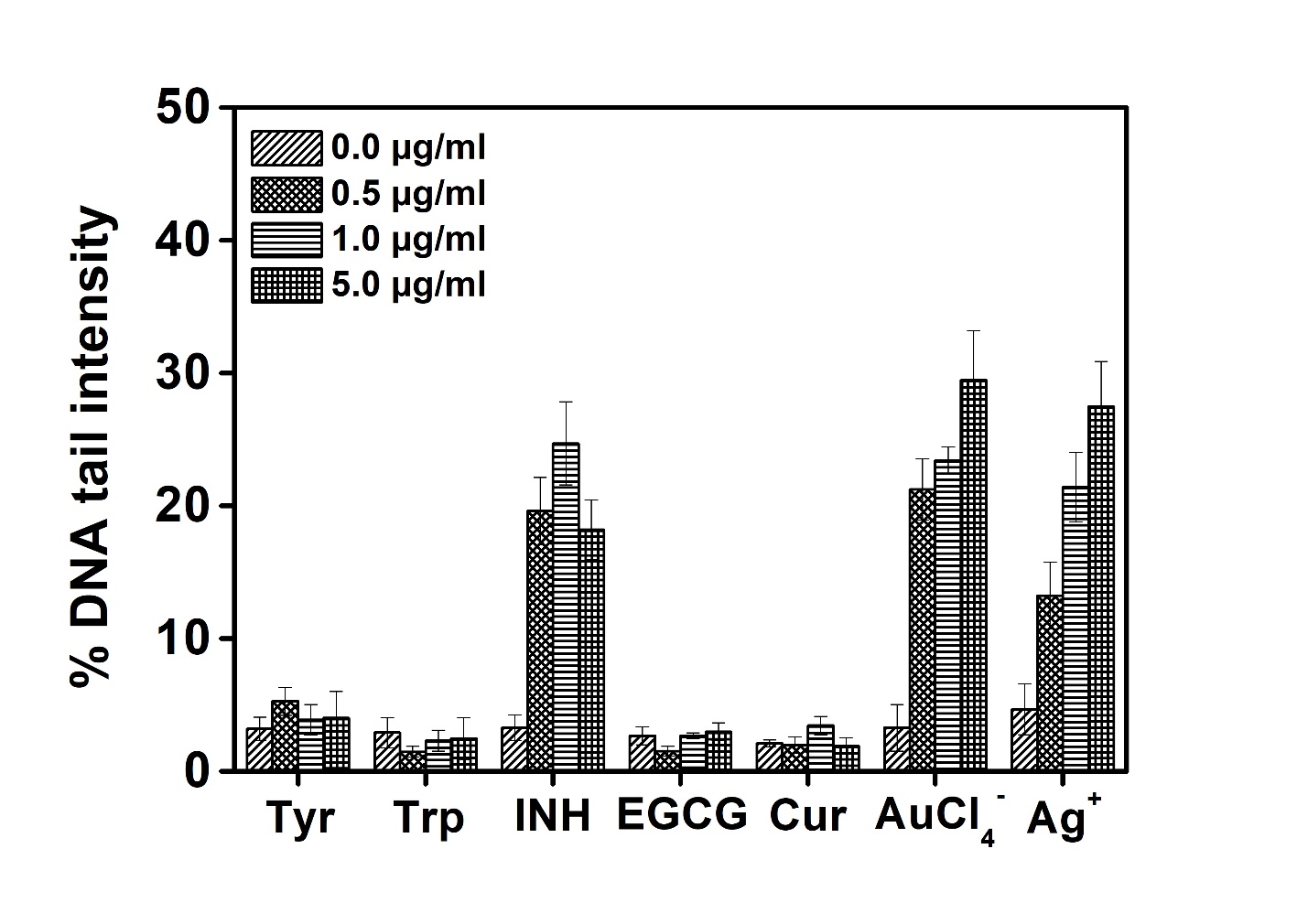
**

**Figure S6:** Genotoxicity (DNA damage expressed as % DNA tail intensity) induced in m5S mouse skin fibroblasts after 24 h of exposure to Tyr, Trp, INH, EGCG, Cur, AuCl_4_^-^ and Ag^+^, respectively.


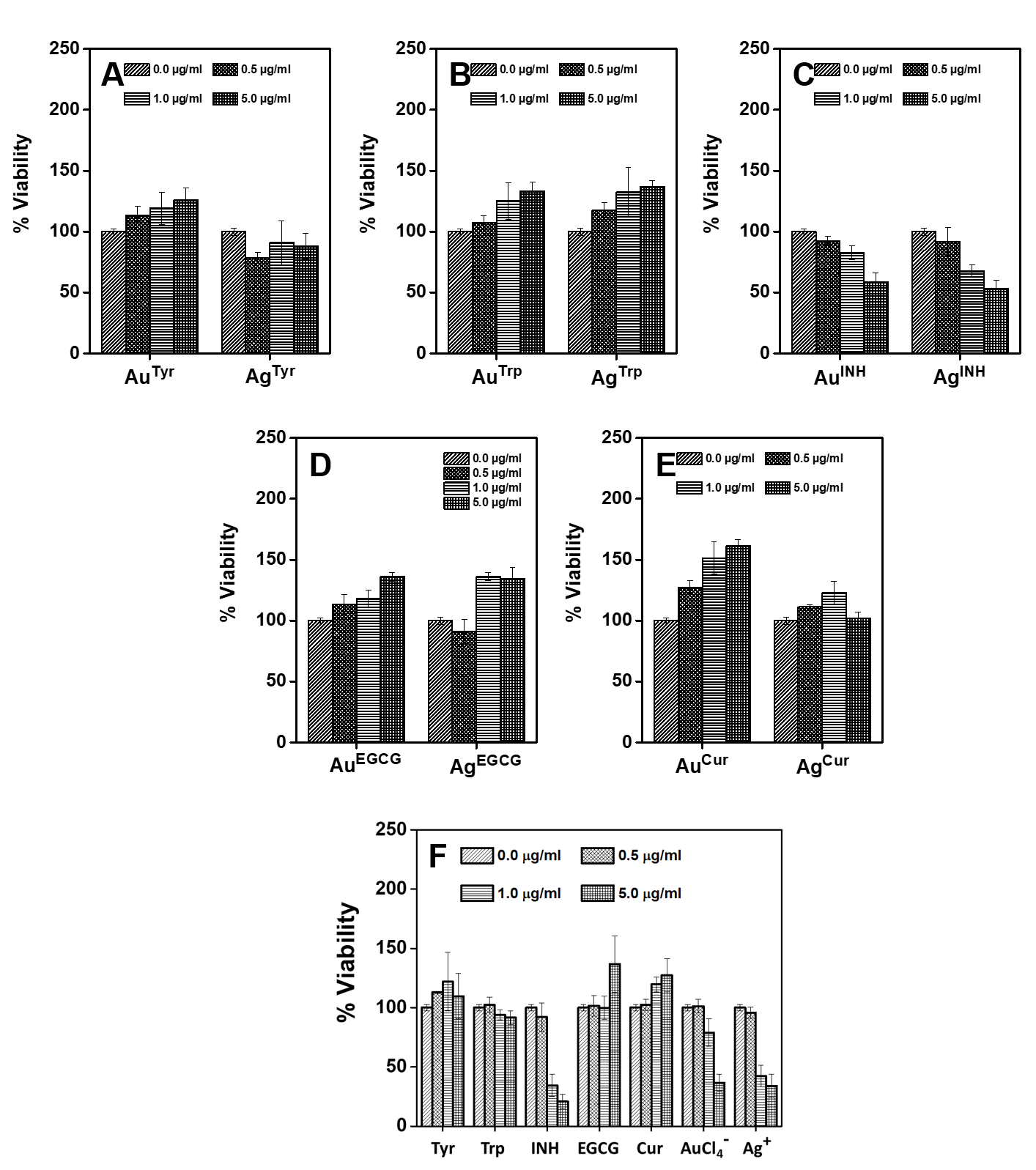


**Figure S7:** Effect of Au and Ag nanoparticles on the viability of RAW-264.7 macrophages. Here, 0.5, 1.0, and 5.0 µg/ml represents metal content in the respective nanoparticles’ solution. The 0.0 µg/ml depicts the control, wherein an equal amount of Milli-Q water was added without any nanoparticle (Panel A-E). Cell viability assessment against Tyr, Trp, INH, EGCG, Cur, AuCl_4_^-^ and Ag^+^, is shown in Panel F. Data represent mean ± SD (n=3).


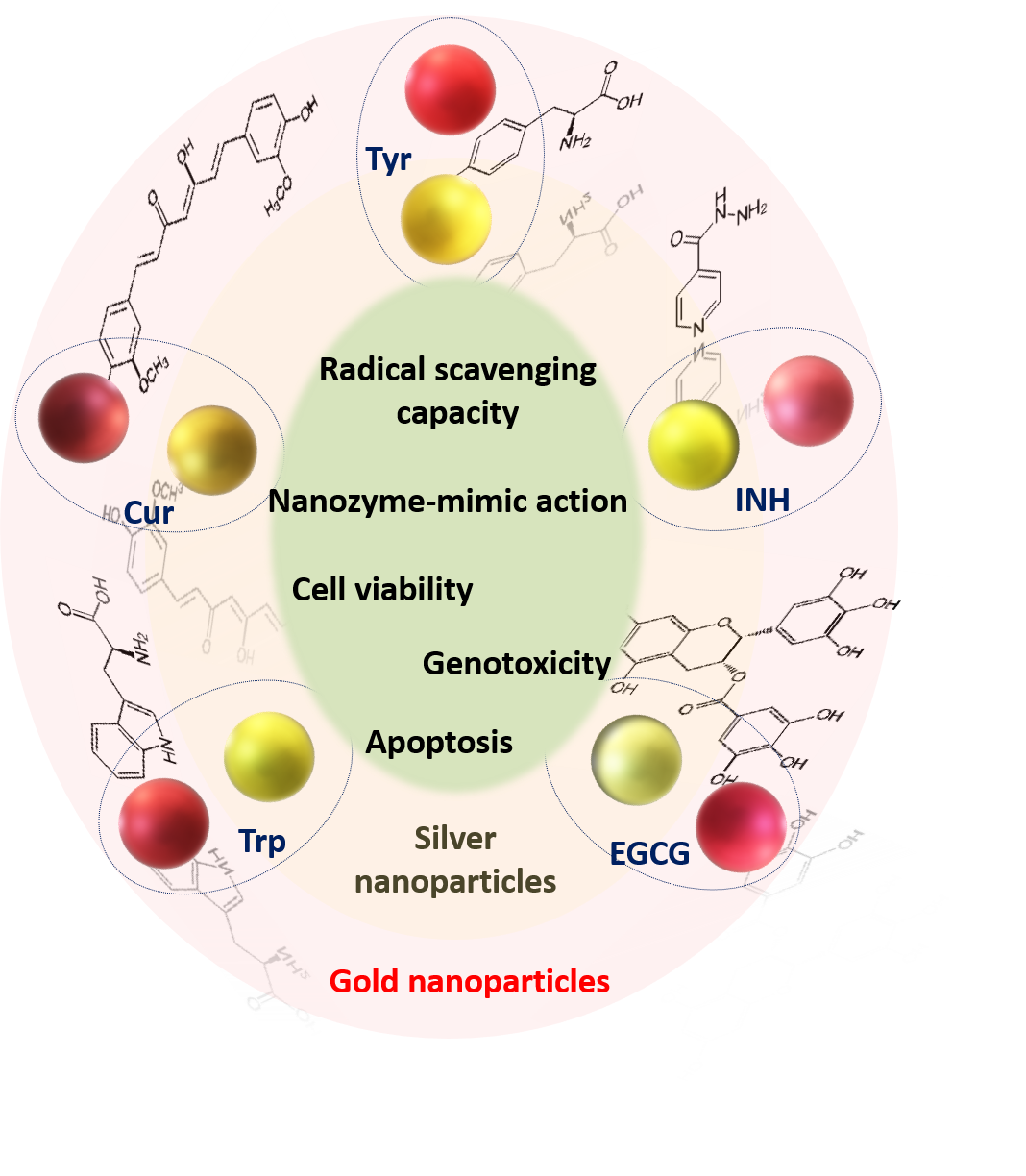


**Figure S8:** Schematic showing the synthesis of Au and Ag nanoparticles with active biomolecular surfaces based on amino acids (tyrosine and tryptophan), antibiotic (isonicotinylhydrazide), and plant derivatives (epigallocatechin gallate and curcumin). Experiments with mouse skin fibroblasts and macrophages demonstrate that the surface corona and metal composition influence the genotoxicity of the nanoparticles.
